# Environmental controls, oceanography and population dynamics of pathogens and harmful algal blooms: connecting sources to human exposure

**DOI:** 10.1186/1476-069X-7-S2-S5

**Published:** 2008-11-07

**Authors:** Julianne Dyble, Paul Bienfang, Eva Dusek, Gary Hitchcock, Fred Holland, Ed Laws, James Lerczak, Dennis J McGillicuddy, Peter Minnett, Stephanie K Moore, Charles O'Kelly, Helena Solo-Gabriele, John D Wang

**Affiliations:** 1 NOAA Center of Excellence for Great Lakes and Human Health, Great Lakes Environmental Research Laboratory, Ann Arbor, MI, USA; 2 Pacific Research Center for Marine Biomedicine, University of Hawaii, Honolulu, HI, USA; 3 Pacific Northwest Center for Human Health and Ocean Sciences, University of Washington, Seattle, WA, USA; 4 Oceans and Human Health Center, University of Miami, Key Biscayne, FL, USA; 5 NOAA Center of Excellence in Oceans and Human Health, Hollings Marine Laboratory, Charleston, SC, USA; 6 Woods Hole Center for Oceans and Human Health, Woods Hole Oceanographic Institution, Woods Hole, MA, USA; 7 NOAA West Coast Center for Oceans and Human Health, Northwest Fisheries Science Center, Seattle, WA, USA

## Abstract

Coupled physical-biological models are capable of linking the complex interactions between environmental factors and physical hydrodynamics to simulate the growth, toxicity and transport of infectious pathogens and harmful algal blooms (HABs). Such simulations can be used to assess and predict the impact of pathogens and HABs on human health. Given the widespread and increasing reliance of coastal communities on aquatic systems for drinking water, seafood and recreation, such predictions are critical for making informed resource management decisions. Here we identify three challenges to making this connection between pathogens/HABs and human health: predicting concentrations and toxicity; identifying the spatial and temporal scales of population and ecosystem interactions; and applying the understanding of population dynamics of pathogens/HABs to management strategies. We elaborate on the need to meet each of these challenges, describe how modeling approaches can be used and discuss strategies for moving forward in addressing these challenges.

## Introduction

Pathogens and harmful algal blooms (HABs) can be significant threats to human health through their presence in drinking water supplies, seafood, and coastal waters used for recreation. Predicting their presence in aquatic environments and quantifying the risk to human health are major scientific challenges that require an understanding of the biological and chemical interactions that control growth and toxicity, as well as the pathways over which they are transported from sources to regions of potential human exposure. Coupled physical-biological models, which aim to accurately represent population dynamics and hydrodynamic transport, are effective tools for studying these interactions, predicting outbreaks of toxic organisms, enhancing understanding of the system and assisting in the management of aquatic resources. Here, we identify three scientific challenges to applying models of biological and environmental controls of pathogen and HAB concentrations and their subsequent impacts on human health:

1) Predicting concentrations and toxicity of pathogens and HABs along transport pathways between sources and locations where they may pose a risk to human health.

2) Identifying the space and time scales at which pathogens and HABs interact with their environment and the appropriate scales at which their concentrations should be sampled or predicted in order to effectively assess risks to human health.

3) Integrating predictions of pathogen and HAB concentrations and toxicity with assessments of human health risks, economic impacts and management strategies.

All of these challenges require integration of modeling with both field and laboratory observations over a broad range of disciplines including aquatic biology, chemistry, ecology, and hydrodynamics. Observations are required to constrain parameters used in models and to validate model predictions. Conversely, models can inform the acquisition and analysis of observations by providing a broader context from which to interpret necessarily limited data collection. Models are also useful in gaining a better mechanistic understanding of the system and its processes. An important goal is to use integrated modeling and observations to test potential management strategies for HABs and pathogens and assess their risk to human health.

## Discussion

### Predicting the concentrations and toxicity of pathogens and HABs

The sources of infectious pathogens in the aquatic environment are varied and can have both animal and human origins. Sick individuals may shed microbes, which are released into the environment through common external sources such as sewer outfalls, wastewater treatment plant overflows, leaky septic tanks, agricultural runoff and at beaches. These pathogens can be subsequently ingested by a healthy individual, thereby propagating the disease transmission cycle. Factors affecting the concentration and toxicity of pathogens include distance from the source, predation, dilution, removal by deposition, regrowth and mortality which can be a function of salinity, temperature, UV light, and other factors. Pathogens known to be a risk to human health include *Vibrio cholerae*, *Vibrio vulnificus, Giardia lamblia, Cryptosporidium parvum*, norovirus, hepatitis A and enterovirus, among many others. Pathogens generally aren't measured directly, but are approximated by indicators of fecal contamination such as enterococci, *Escherichia coli *and fecal coliforms. It is known that such indicator organisms may respond to these factors differently than the pathogens themselves. See Stewart *et al*. (in this supplement) [1] for more information on the sources and effects of pathogens.

Toxic HAB species are indigenous to many aquatic systems, but may be dispersed through ballast water, oceanic currents, birds and other wildlife [2,3]. While not all marine and freshwater algal genera are capable of toxin production, those that do may adversely impact human health when they reach bloom concentrations, either through direct consumption of water, inhalation of aerosolized particles, skin irritation during recreational contact or through accumulation in shellfish and/or finfish. Factors impacting the concentration and toxicity of HABs include nutrient concentrations, light, hydrodynamic mixing, strain composition, life cycle transitions and presence of grazers and/or competition [4]. Erdner *et al*. (in this supplement) [5] provide more detail on HABs that impact human health.

The multitude of factors that can impact the severity of HAB and pathogen outbreaks calls for the use of models to aid in the prediction of potential effects on human health. These predictions require an understanding of the forces that affect the initial concentrations, how environmental conditions impact growth and toxicity/pathogenicity and how the concentrations of pathogens and HABs change during transport. Forces that act at a number of different spatial scales impact these processes. For example, at scales on the order of the cell diameter, growth of a planktonic species is a function of the diffusion-limited supply of substrates to the cell membrane and the physiological capability of a cell to take up and assimilate nutrients. The mortality of a species depends in part upon losses to predators, which are a function of the concentrations of the predator and prey, the encounter rate of the two populations, and the efficiency with which the predator harvests the prey. On a larger spatial scale, mesoscale physical processes such as currents, eddies, convergences and upwelling zones all can potentially influence the distribution of HABs and pathogens [3,6,7]. Knowledge of organism abundance is not necessarily sufficient because production of the harmful compounds depends on complex intracellular processes that can cause cellular toxicity/pathogenicity to vary by orders of magnitude. The genetic diversity of organisms is also an important factor in predictive modeling [8]. In other words, equal numbers of organisms can have very different overall toxicity. Both the population dynamics of the organism and its toxin/pathogen production can be modulated in a variety of ways by environmental conditions such as temperature, salinity and nutrient availability; all of which can be influenced by human activities in a variety of ways. Human activities can also complicate matters by introducing substances into the environment that may affect growth and toxin production, including organic and inorganic chemicals such as polycyclic aromatic hydrocarbons, pesticides, herbicides, and brominated flame retardants; many of the interactions and impacts of these compounds are still relatively unknown.

#### Conceptual underpinnings of model formulation

**Figure 1:**
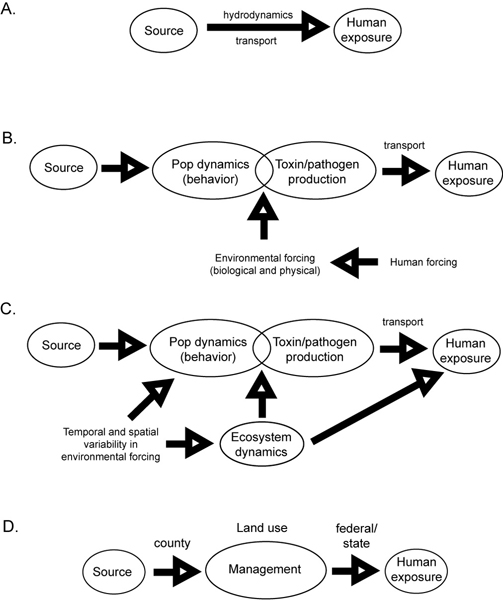
Modeling approaches for linking sources of HABs and pathogens in the oceans and Great Lakes to human health.

Modeling approaches are a means by which to address these varying components in connecting sources to human exposure. There are several layers of complexity in this process (Figure 1). In the simplest case (Figure 1A), currents disperse these agents from source regions to areas in which human activities can be impacted. Even this simplest case is challenging because neither the sources nor the transport pathways are typically well characterized and can be complex within turbulent coastal systems that have time-varying and spatially-dependent forcing. In addition, the source cannot always be treated as persistent in space or time, primarily because of the biological and physical processes that regulate its expression, as described above (Figure 1B). Although the dynamics of the causative organisms can sometimes be effectively isolated from the rest of the ecosystem, such is not always the case (Figure 1C). The need to explicitly resolve ecosystem dynamics is especially acute when the causative organism constitutes a significant component of the pathogen/algae community, as often occurs in harmful algal bloom phenomena. In such circumstances, the organism can have direct impacts on both bottom-up controls such as nutrient/light availability and top-down controls via grazing. Therefore, since the conceptual frameworks underpinning these various approaches to pathogen and HAB problems differ widely in their level of abstraction, the observational and modeling strategies needed to address these issues must be tailored to each application, and its assumptions, accordingly.

#### Applying modeling approaches

The range of models used in simulating the fate, transport and concentration of HABs or pathogens is wide and depends on data availability as well as the level of mechanistic understanding. At one end of the range, empirical models are focused on a few controlling parameters and the dependent variable, e.g. the concentration of a HAB or pathogen. These models rely on statistical relationships between observed parameter (e.g. environmental conditions or indicator organisms) with the pathogen or HAB concentration to determine the reliability, sensitivity or specificity of a given parameter for prediction [9,10]. While easily derived, these relationships are likely to be site specific. Coupled biological/physical numerical models link a hydrodynamic model that simulates transport and dispersion with a mathematical model describing ecosystem dynamics and the response of the HAB species or pathogen to environmental controls (Figure 1). These models can be very complex and require a considerable amount of data. For example, a pathogen fate and transport model may incorporate functions to account for microbial sources, die-off, deposition, regrowth [11,12] and (in some cases) impacts to shellfish [13]. Models of this type are often adaptable to different locations and changing environmental factors driving the system and can therefore be useful as predictive tools.

Models generally require significant amounts of data to make accurate predictions. However, even models that are well supported by data are subject to uncertainty because our knowledge of ecosystem dynamics, hydrodynamic transport and dispersion processes will always be incomplete. Uncertainty in model input variables such as initial conditions, sources and environmental forcing factors, combined with the inability of models to resolve all the spatial and temporal scales of variation, results in uncertainty in model predictions. Even once there are well-established models, the connections between the populations of pathogens or harmful algae and prediction of human health impacts are not straightforward.

**Figure 2:**
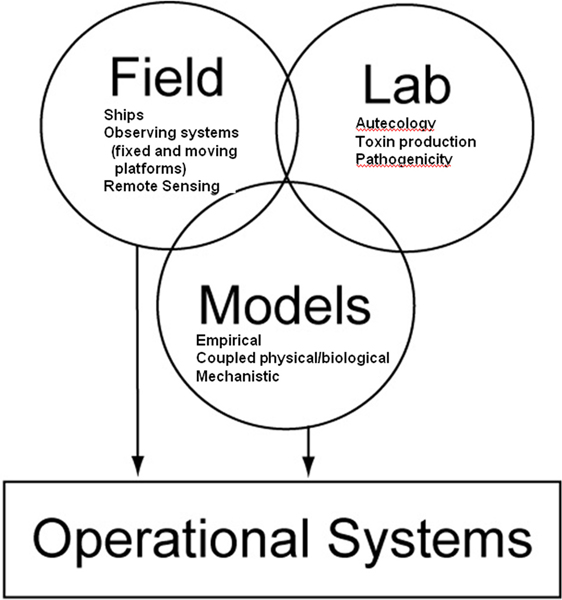
This diagram illustrates the three components that are key to connecting pathogen and HAB populations to operating systems that predict human health impacts: field work, laboratory experiments and modeling.

The predictive capability of a model can be described in terms of accuracy and specificity. Accuracy is the ability of the model to predict the endpoint of interest whereas specificity is the ability of a model to be selective for the outcome of interest. In practice, this quantitative assessment can be carried out with formal validation approaches and/or reiterative evaluation. Models are validated in several ways. Dependence of the solution on input parameters can be quantified by systematic variation of those parameters about a baseline case. Simulation studies can help in this identification and can generate new predictions. Direct testing of field observations and/or laboratory studies against model predictions is a way to test the predictive capability of models and the resulting inconsistencies help to identify gaps in our understanding of the ecosystem. While such comparisons are often limited due to the necessarily smaller spatial and temporal scale of observational surveys compared to typical model domains of interest, modeling approaches can provide significant insights with respect to the factors that influence HAB and pathogen concentrations in coastal ecosystems and further develop a more mechanistic understanding of not only if, but *how *these factors have an impact. The integration of *field work *(to identify what environmental controls, hydrodynamic forcing factors and population dynamics are associated with HAB or pathogen presence and toxicity), controlled *laboratory experiments *(to determine if those associations are significant and quantify parameters required for biological models) and *modeling *(to inform future sampling, prediction and forecasting) is the key to connecting pathogen/HAB populations to human health impacts (Figure 2).

Modeling approaches that integrate field and laboratory data have been used successfully to predict concentrations of pathogens and HABs in aquatic systems, as demonstrated in the following series of examples. The closing of swimming beaches due to potentially high levels of pathogens, as assessed by indicators of fecal contamination, is an excellent example of the need for new modeling approaches. Under current regulations, beach closures and advisories are issued when indicator microbe levels exceed a specific threshold. Beach closings are costly, inconvenient and, at times, unnecessary. Unnecessary beach closures occur for two predominant reasons. First, the sporadic nature of the exceedences is not compatible with the long analysis times that most traditional monitoring methods require (e.g. on the order of 24 hours). For cases where the sources of indicator bacteria are intermittent (on time scales less than days), the monitoring program is incapable of providing warnings in a time effective manner. By the time the warning is in place, the bacterial levels may no longer be high enough to require a beach closure. Beach closures are a significant issue in the Great Lakes region, where there are over 500 beaches used for recreation. Research on one such beach along the Indiana coast of Lake Michigan (Burns Ditch, Portage, Indiana), has shown that water from this source contributes significant amounts of *E. coli *and associated bacteria to the near shore beach areas of southern Lake Michigan, but the current beach monitoring program has been proven inadequate, particularly if samples are collected only periodically. To protect human health among beach users in this area researchers have developed a coupled model that incorporates models of near-shore hydrodynamics, hydrological input, near field mixing, pathogen loading (through tributaries, non-point sources and resuspension), and bacterial fate and transport in order to assess the need for beach closures in this area within time scales consistent with the sources of indicator microbes [12]. This type of coupled model could also be applied to protect human health from the impacts of pathogens on drinking water supplies, such as those experienced by the city of Milwaukee in 1993 when an outbreak of *Cryptosporidium *caused illness in 400,000 residents [14]. There were multiple factors affecting the severity of this event, including heavy precipitation increasing pathogen loading, transport of pathogens in nearshore Lake Michigan from the wastewater outfall to the drinking water intake, and survival rates of the pathogens in the process of transport and treatment.

Another primary cause of unnecessary beach closures is associated with the unknown relationship between health effects and indicator microbes which come from non-point sources. Non-point sources can include humans, animals, and potentially regrowth within shoreline sediments [15,16]. As a result of uncertainties in the ability of indicator microbes to track human health effects in areas impacted by non-point sources, models should focus on simulating pathogen sources specifically, which are considered to be a better indicator of human health effects. Pathogens may also be present in the absence of elevated indicator levels due to the ability of some pathogens to survive treatment or otherwise persist longer than indicator bacteria, or be present at low but dangerous concentrations following dilution effects.

Pathogen concentrations at a beach are controlled by both transport of pathogens to the beach and their fate once there. For example, many studies have shown that the indicator microbe, enterococci, can grow in the sand at the waters edge and from there can be suspended into the water column. To account for this *in situ *growth, a pathogen fate and transport model was coupled to a hydrodynamic model and was parameterized to simulate enterococci at a 1.6 km stretch of beach in Miami, Florida [17]. This model, still under development, employs a fixed finite element triangular grid in which the tidal oscillation of the water surface successively dries and wets the computational cells in the intra-tidal zone, simulating the back-and-forth movement of the waters edge and the zone of microbe re-suspension. The inactivation of enterococci by phototoxicity is modeled as a first order decay process with the decay coefficient dependent on solar radiation and calibrated to experimental data. Since the site is a protected bayside beach with prevailing offshore winds from the east, wind waves are modeled only as a microbial load re-suspension mechanism through their stirring of the beach sand containing microbes at the water's edge. The wave surf circulation is very weak and is ignored. Others have modeled beaches where the wave action is stronger and is thus important in diluting the microbial concentrations [17,18]. Pathogen sources from animal (i.e. dog and bird) use of the beach, as well as human bather shedding have been quantified through field experiments and digital camera monitoring of the beach provides information on timing, location, and quantity of potential sources. Through model experiments, expected variations in water column microbe counts can be quantified for each of the source functions or combinations of functions to supplement the information obtained from the field monitoring. To date, the model has been used to study loading processes, however, due to sparse field observations, actual hindcast or predictive simulations have not yet been made.

In another example, a modeling approach has been successfully used in predicting harmful algae concentrations. Ciguatera is the most common agent of seafood poisoning in the world; it is estimated to account for 95% of the medical costs associated with harmful algal blooms. Humans acquire ciguatoxin by eating reef fish that have accumulated the toxins originally produced by the epiphytic dinoflagellate *Gambierdiscus toxicus*, and biomagnified and biotransformed via the marine food web. The factors controlling *G. toxicus *abundance and/or ciguatera outbreaks are poorly understood. In order to better predict variations in *G. toxicus *abundance due to variations in environmental control parameters, a model was developed using smoothed time-series abundance data from regular sampling along the coast of Hawai'i and literature-based kinetic constants for *G. toxicus *populations from around the world (M. Parsons, pers. comm.). The *Gambierdiscus *time-series generally parallels that of ciguatera incidents in Hawai'i. Application of such constants to the simulation model is intended to improve the understanding of the dynamics of *in situ *toxin production leading to ciguatera poisoning.

In New England, the most serious HAB issue is paralytic shellfish poisoning (PSP), a potentially fatal illness that occurs when humans eat shellfish that have accumulated saxitoxins from dinoflagellates in the genus *Alexandrium *[19]. Linkage between the population dynamics of *Alexandrium *and the hydrodynamic environment arises from three basic sources: ambient water properties, spatial distributions of populations as influenced by ocean currents and the swimming ability of *Alexandrium*. Coupled physical-biological models offer a framework for diagnosis of these manifold contributions to variability in *Alexandrium *populations in the Gulf of Maine (GOM). Extensive comparisons between simulated and observed physical and biological fields suggest that the model is capable of capturing many aspects of the temporal evolution and spatial distribution of a particularly severe bloom that occurred in 2005 [20]. Hindcast sensitivity experiments based on the 2005 data distinguished the roles of three major factors hypothesized to contribute to the bloom: 1) the high abundance of newly deposited *A. fundyense *cysts in western GOM sediments; 2) strong northeaster storms with prevailing downwelling-favorable winds; and 3) a large amount of fresh water entering the GOM due to abundant rainfall and heavy snowmelt. Newly deposited cysts in the western GOM appear to have been the primary causative factor of the 2005 bloom [21]. Wind forcing was an important regulator, as episodic bursts of northeast winds caused onshore advection of offshore populations. These downwelling-favorable winds also accelerated the alongshore flow, and anomalously high river runoff in 2005 resulted in stronger buoyant plumes/currents, both of which resulted in transport of high cell concentrations into Massachusetts Bay. This work has shown that coupled hydrodynamic/population dynamics models of this type may be able to forecast large scale seasonal characteristics of the bloom.

### Space and time scales of ecosystem dynamics and environmental monitoring

The abundance of organisms such as harmful algae, pathogens and microbial indicators are heterogeneous because they are influenced by physical, chemical, and biological processes that vary across a wide range of spatial and temporal scales in the aquatic environment. This variability, which is inherent in the distribution of organisms, presents challenges to investigators who seek to model abundance and growth in relationship to environmental conditions and sample appropriately. Natural spatial and temporal variability provides a heterogeneous background that makes it difficult to understand and quantify ecosystem responses to specific stressors. For example, natural changes in water quality that occur at different time scales (e.g. seasonally or tidally) may be so large as to mask changes in water quality that occur as a result of human activities [22]. Random or episodic events that are not accounted for in the sampling approach may introduce additional variation or noise leading to inappropriate management decisions and actions [23]. This heterogeneity may reflect simple environmental gradients (e.g., distance from pollution sources, salinity change, and sediment type differences) or more complex processes such as seasonal successional changes or complex ecological interactions. Human influences and linkages to the aquatic environment also vary in space and time and can interact with natural processes to create intricate and perplexing patterns. Failure to understand interactions between natural processes and change due to human activities frequently makes it difficult to link specific sources of HABs and pathogens to changes in ecosystem and public health.

#### Spatial and temporal scales expressed in Stommel diagrams

**Figure 3:**
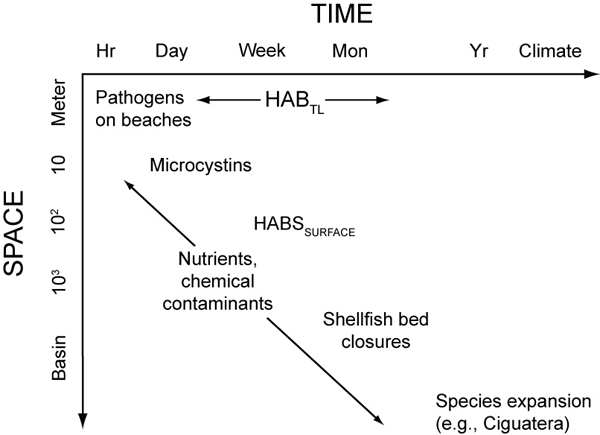
Examples of the characteristic temporal and spatial scales utilized in sampling aquatic organisms that affect human health. The influence of nutrients and chemical contaminants extend across all scales in space and time. This schematic is a simplified representation of spatial and temporal scales of interest, as illustrated by the distinct scales for Harmful Algal Blooms in thin layers (HAB_TL_) and in extended surface blooms (HAB_SURFACE_).

The spatial and temporal dimensions of this variability can be expressed as schematic plots that are often referred to as "Stommel" diagrams. Here we illustrate the relevant space and time scales for some of the aquatic organisms that affect human health (Figure 3). The time scales range from days for beach closures, weeks to months for HABs [24] and years to decades for cholera outbreaks. The corresponding spatial scales at which aquatic organisms affect human populations increase proportionately from hundreds of meters at beaches [25] to up to 1000 km for cholera [6].

Beach closures associated with high counts of fecal coliforms occur at 'event' scales of days with corresponding spatial scales on the order of meters to hundreds of meters. The physical processes that transport pathogens from source regions, such as a watershed or sewage outfall, to a beach include runoff following rain events, tidal currents and buoyant plumes [26,27]. The variability of abundance in source waters, such as rivers in urban environments, can vary from <100 to >1000 organisms per 100 ml, as most probable units, within distances of a few meters [25]. Average monthly counts may also vary by more than an order of magnitude at individual stream stations. If this variability is characteristic of riverine water discharging into the coastal environment, sampling strategies to monitor conditions for beach closures must consider short-term fluctuations in source waters as well as runoff events.

Time scales on the order of weeks to months are characteristic of the duration of HABs. In North American coastal waters, different species of dinoflagellates frequently develop bloom conditions that last for several months in the eastern Gulf of Mexico, Gulf of Maine and along the Pacific coast [7,28]. These blooms extend over distances of tens to hundreds of km at the oceans' surface, while other species, such as *Karenia mikimotoi *and *Dinophysis acuminata*, can exist as layers in the water column with a thicknesses ranging from tens of centimeters to meters [29]. The development of these 'thin layers' complicates the sampling strategy and requires a means of continuously mapping the vertical distribution of organisms with depth.

Horizontal distributions of algal cells within a bloom are also spatially heterogeneous. For example, once a bloom of the toxic dinoflagellate *Alexandrium fundyense *initially develops from benthic cysts in the western Gulf of Maine, the surface distribution is largely regulated by population growth and the transport of cells by surface currents as modified by local winds [7,30]. Regions of high cell density in surface waters of the GOM can exhibit a bifurcated pattern that parallels the flow of surface currents, which impacts, which shellfish beds will be, exposed to the PSP toxins that these cells produce. The patchiness in surface distributions of the toxic dinoflagellate *Karenia brevis *is evidenced by the fact that advisories must be issued daily along the west Florida coast to warn individuals with respiratory problems who might inhale the aerosolized toxins [31,32]. High variability in cell concentration can result in beaches separated by only a few kilometers differing markedly in their suitability for bathers.

The variability in environmental conditions and processes that influence the population dynamics of the target pathogen or HAB has also been demonstrated through a network of automated observing systems in the Hood Canal sub-basin of Puget Sound, Washington. The Oceanic Remote Chemical-optical Analyzer (ORCA) is a moored buoy that telemeters hourly profiles of oceanographic and atmospheric properties in near-real time [33]. The high frequency of observations allows for patterns of environmental variability occurring on hourly (e.g. tides) to interannual (e.g. El Niño/Southern Oscillation) timescales to be resolved and has revealed the high variation of biological variables on various temporal/spatial scales in Puget Sound [34], further demonstrating that the development of algae blooms cannot always be resolved by monthly sampling.

The periodicity in cholera outbreaks in coastal communities exists at temporal scales of years to decades. The pathogenic bacteria *Vibrio cholerae *is widespread in coastal waters and exists in rivers as well as the open ocean. However, the disease is endemic only in subtropical and tropical latitudes with peaks in reported cases occurring in spring and fall in Asia around the Bay of Bengal, and more recently coastal South America. There is a demonstrable association of cholera outbreaks with El Niño conditions [6], leading to the development of a hypothesis that climate-driven events enhance the emergence of virulent strains of *Vibrio cholera *with transmission to humans through marine invertebrates.

#### Linking observations with models

A fundamental problem for investigators is that a mismatch often exists between the scale at which organisms are sampled and the scales at which their growth and distribution are regulated by environmental conditions. Accurate detection of pathogens can be challenging due to their presence in low concentrations and their patchiness in distribution. The paucity of field monitoring data can be mitigated through the use of models to describe the expected spatial and temporal fields under given source concentrations and environmental conditions. Models can also be used to guide sampling effort and are helpful in understanding whether or not the results obtained from a particular sample collected at a particular point in time and space are representative. For example, the typical sample size for routine water quality monitoring purposes is 100 ml, a size that is approximately 7 or 8 orders of magnitude less than the size of the water body to be evaluated for safety. Given the results from a particular sample, the model could thus serve in hindsight to provide information concerning the range of results that could have been predicted if the sample were collected at a set time or distance removed from the original sample. During an epidemiologic study, models can be designed to determine where the expected range of pathogen or toxin levels would be found to best represent conditions for human health exposures.

For beaches with non-point sources of pathogens, the timely warning of health hazards is a challenging objective that remains elusive in part because of the disparity of scales of monitoring and the scales of concentration variation in the environment. At beaches with no known point source of pathogens, regulatory monitoring typically consists of infrequent samples, once a week at one location, for a large transiently shifting volume of beach water stretching thousands of meters along the beach and 30 m offshore. In contrast to beaches exposed to known (relatively remote) point sources, the loading at non-point source beaches may originate locally at the shoreline and/or result from discrete events, such as bather shedding [16] and the impacts to beaches and swimming areas may be influenced by hydrodynamic factors. Again, here time scales are of importance specifically with respect to factors that influence the magnitude and direction of ocean currents and density stratification of the ambient water [35]. A coupled nearfield and farfield model of the Mamala Bay (Oahu, Hawai'i) outfall [36,37] showed extreme variability of the plume behavior due to changes in currents and density stratification with nearfield dilution from one hundred to several thousands. Long-term simulations predicted that flushing, horizontal diffusion, and decay would result in very high dilutions, preventing any significant buildup of microbial contaminants. Simulation results showed that outfall impacts to nearby beaches were considered to be very small, with other non sewage sources such as flows from canals and storm water discharges as the primary contributor to higher levels of bacteria.

The shallow water and moving boundary inherent to beaches are not handled well by present models that use fixed computational grids [38]. These models are also mostly designed to simulate water column constituents as part of the continuum. The continuum approach is adequate when there are sufficient particles spread out in a computational cell to allow for characterization of the mass present with a concentration value for that cell. However, in reality indicator microbes and pathogens may act more as separate particles possibly occurring in small patches due to the discrete character of the sources in time and/or space, so other ways of characterizing their distribution are needed. Presently one way of overcoming this is to use Lagrangian tracking of individual particles or patches. However, for large numbers of particles the computational burden becomes excessive and the physical processes down to the scales that need to be resolved may still be inadequately represented. Ensemble forecasts, perhaps similar to techniques used in weather forecasting, may also be used to address such sparse or patchy distributions of microbes. The interaction of microbes or pathogens with solid particles that are suspended into the water column or settle to the bottom at different times (for example, over a tidal cycle) and locations could be important but has received little attention. In some circumstances, coupling of the water column hydrodynamic and ecosystem models to a sediment model may be essential to accurately represent the relevant interactions.

Field data on the transport of blooms can advantageously be incorporated into models. Off the coast of Washington State, drifters have been employed to track the transport of blooms of *Pseudo-nitzschia *to coastal razor clamming beaches. The diatom *Pseudo-nitzschia *produces the toxin domoic acid, which accumulates in bivalves and may cause human consumers a potentially life threatening illness called amnesic shellfish poisoning [39]. Drifters were deployed in the Juan de Fuca eddy, a highly retentive oceanographic feature and initiation site for blooms, and tracked via satellite. Drifters that escaped the eddy were advected southward in the coastal jet during periods of upwelling favorable winds. In order for blooms to impact coastal beaches, research has shown that a storm with downwelling favorable winds is necessary to drive onshore transport [3,40]. Drifter locations are accessible in real-time by managers to assist with timely decisions about the safety of shellfish harvest at coastal beaches.

The use of data assimilation, a technique by which model simulations are repeatedly adjusted during a simulation with observations at discrete locations and times, has in some cases significantly improved model skill. Formal methods of data assimilation offer a variety of means to constrain coupled physical-biological models with observations [41]. The last two decades have seen a dramatic increase in the types of data that are input into such models, and the development of robust and varied approaches for assimilation [e.g. [42-45]]. For instance, data assimilation can reduce model-data misfit by recovering optimal parameter sets using multiple types of data [46,47]. Perhaps even more importantly, these data assimilation analyses can demonstrate whether or not a given model structure is consistent with a specific set of observations. When model and data are shown to be consistent, the specific mechanisms underlying observed patterns in simulated distributions can be identified.

Growing needs for real-time information concerning ocean processes relevant to human health have brought the issues of model prediction and forecasting to the forefront. However, it is clear that much more work needs to be performed before this becomes a realistic and achievable goal. One of the first applications of data assimilation within marine and atmospheric science was in atmospheric weather modeling and forecasting and the technique is particularly helpful in complex systems where continuous data records are available at one or multiple locations. As a similar abundance of high resolution biological and chemical data become available for the ocean and coastal waters, by means of satellite remote sensing and ocean observing systems, these techniques can also be advantageously employed in future pathogen and HAB modeling. Until this time, and until a better understanding of the dynamics of marine systems is attained, data assimilation in coupled physical-biological models will be likely to be used more for model improvement and parameter estimation than for operational prediction. A necessary precursor to the latter is the quantitative demonstration of forecast skill in specific applications [48].

Models do not solve problems of scale mismatching, rather the selection and use of a model (for example choice of grid resolution, or choice of algorithms) implicitly specify the scales that will be represented in solutions. By validating model results against observations, issues of a scale mismatch will appear as inability (or poor ability) to properly describe certain observed features. Typically, it is the small features that are poorly represented or absent in models because of the need to limit the smallest grid size based on computational efficiency. Modeling approaches can be used to evaluate the most appropriate scale over which to sample and predict pathogen and HAB concentrations based on their variability in time and space. In particular, observing system simulation experiments (OSSEs) offer a means for quantitative evaluation of sampling design. This technique has its origins in dynamic meteorology [49] and is recognized as an important tool for the development of oceanographic sampling systems [50-53]. The approach begins with the construction of a simulation that is characteristic of the natural system. The model run serves as a space/time continuous representation of reality, which is then subsampled in a specified fashion to produce a simulated data set. The simulated data are then fed into an analysis scheme in which they are synthesized into a reconstruction of reality. Comparison of the reconstructed field with the "truth" as defined by the original simulation thus provides a quantitative evaluation of that particular sampling strategy and the associated analysis scheme. Of course there is an important caveat to such an evaluation: the OSSEs are based on simulations that are imperfect representations of the natural system. Thus, care must be taken to restrict the scope of the OSSEs to aspects of the model that are realistic. The key to successful modeling is to critically examine model results against observations and to make necessary modifications in order to achieve a proper match of scales between model simulations and dominant prototype processes.

### Connecting HABs and pathogens to human health risks and resource management

In order to connect the understanding of the dynamics of HABs and pathogen populations and the ecosystem within which they live to the effects on human health, it is probably most useful to work backwards along the pathway indicated in Figure 1. The rationale is that working backwards from human health effects ensures that the modeling process is focused on the human health endpoint. Following this logic, the first step in the modeling process is to relate exposure to human health consequences. This falls within the realm of epidemiology. Epidemiological models are typically probabilistic, that is, a specified degree of exposure is associated with a certain probability of some human health endpoint [54]. Death is one endpoint, but non-lethal effects are also of great concern. Thus a specified degree of exposure may be associated with a variety of endpoints, each with its associated probability. One complicating factor is the duration of exposure. Chronic exposure will generally produce adverse health effects at substantially lower doses than acute exposure.

Simplistically, what is needed for an epidemiological model is dose and response information, but this is seldom available or directly obtainable if the endpoint is a human health effect. The mechanism of exposure often, but not always, involves ingestion. If the mechanism of exposure involves oral ingestion of water, the model will be probabilistic for recreational exposure, since not all persons who go to the beach swallow the same amount of water. If the mechanism of exposure involves entry through cuts and abrasions, the model will require information on the percentage of at-risk bathers and their degree of susceptibility to infection. In some cases the mechanism of exposure involves inhalation, in which case the degree of exposure depends on the distribution of people downwind of the source. In the case of fish and shellfish consumption, the input to the exposure model will require information on the concentration of the toxin in fish and shellfish consumed by the at risk population. Assuming that the food chain is the pathway by which the toxin finds its way into fish and shellfish consumed by humans, input to this model will require information on the concentration of toxin in the microbes in question and the concentration factor between the microbes and the relevant fish and shellfish. This sort of information may come from controlled experiments with captive animals or from sampling of natural populations. Due to time lags between the production of toxin by microbes and the appearance of those toxins or their metabolites in edible fish and shellfish, developing a realistic concentration model is a difficult task. The problem will be confounded in the case of fish, which are motile, and hence may be sampled at locations distant from the original source of toxin production.

#### Applying modeling approaches

Models are useful for management purposes for two primary reasons. First, models provide managers with the ability to assess the impacts of different sources on HABs and pathogens within receiving water bodies. Managers can use models to evaluate the impact of suspected sources by simply removing those sources from the model [55]. Such a concept is manifested within the regulatory framework by the establishment of Total Maximum Daily Loads (TMDL) for a particular watershed. A peculiar management policy that confounds the inclusion of sources and exposure in a management model (Figure 1D) is that exposure is typically the purview of federal and state entities, whereas regulatory actions concerning sources (such as land use practices) are handled at the county level. Second, models are valuable tools for management purposes because they can be used in a predictive forecasting mode, for beach advisories or toxic algal bloom development. Integrated observation and modeling systems are capable of accomplishing what monitoring alone could never accomplish, even in the ideal scenario of real-time measurements, which is warning the public ahead of time when health risks are present. Routine sampling and analysis should continue, nevertheless, as such data should be used to continuously validate and verify the model.

Beach closures due to unsafe levels of domoic acid in shellfish tissues from the harmful algal bloom *Pseudo-nitzschia *species are relatively recent phenomena in Puget Sound (WA), with the first closure documented in 2003 [56]. Subsequent closures were documented in 2005 [57], suggesting that domoic acid may continue to threaten shellfish harvest areas in the future. Grazing rates of 13 common shellfish species on *Pseudo-nitzschia *and detailed information on beach profiles and species assemblages in northern Hood Canal, Puget Sound, are being incorporated into an advection-diffusion model to determine the likelihood of acute contamination of the nearshore shellfish community and the potential risk to human harvesters and consumers. Such values are especially important for accurate prediction of contaminated shellfish as there can be dramatic differences in toxin retention between shellfish types (e.g. blue mussels retain domoic acid for days versus razor clams retain the toxin for months.)

Another way in which modeling has been used to guide management decisions related to oceans and human health is exhibited in the use of tidal creek watersheds as important habitats for identifying and obtaining early warning of environmental and public health issues associated with rapid southeastern coastal development. In the southeastern U.S., tidal creek watersheds are among the most rapidly developing regions in the nation. Tidal creeks are the primary interface between the landscape and estuaries where freshwater from the land mixes with saline water, resulting in dynamic environments that are renowned for their ecological complexity, biological productivity and seafood production [23,58]. As the first zone of impact for non-point source pollution runoff, the potential for the microbial and chemical contamination in tidal creek habitats is great. Based on the data collected by Holland et al. [23], a conceptual source-receptor model was developed. This model provides an overview of the linkages between the stressors associated with non-point source runoff from coastal development and the ecosystem and societal responses that result, including shellfish bed closures from pathogen contamination and the flooding vulnerability of adjacent uplands during storm events. The elements of the model have been validated for South Carolina, North Carolina, and Georgia, and have proven useful for communicating results to general and technical audiences.

## Conclusion

In this merging of science and society, modeling approaches can be of great assistance in meeting the challenges of identifying and predicting the impacts of HABs and pathogens on human health. Making the link from sources to human health requires the ability to predict concentrations and toxicity/pathogenicity, identify the appropriate space and time scales over which to measure and model, and integrate model predictions with assessment of human health risk and management strategies. Key to the effort to connect sources of HABs and pathogens to impacts on human health is the ability to integrate field and laboratory studies into modeling efforts: to use empirical observations from the field and mechanistic understanding from the laboratory to provide input to the models and also use modeling output to guide sampling efforts and point out the gaps in the mechanistic understanding of the system. Opportunities to share resources in terms of field data, important physical and ecological processes and to synthesize different modeling approaches will significantly advance our understanding of these systems and improve our predictive capabilities. Collaboration between researchers from multiple disciplines as well as engagement between empiricists, modelers and stakeholders will be necessary to develop and apply modeling approaches in a way that truly can connect sources of HABs and pathogens to human health in a meaningful way. There have been many successes in using modeling approaches to inform management decisions to protect human health and interdisciplinary and continued development of collaborative research is essential to continue to address the challenges that remain.

## Competing interests

The authors declare that they have no competing interests.

## Authors' contributions

JD coordinated the manuscript. DJM and JD co-chaired the session at the NSF/NIEHS Oceans and Human Health meeting at WHOI that formed the basis for this manuscript. All authors contributed text and revisions, with especially significant contributions by JL, HS-G, and JDW. Figures were contributed by DJM, JD, EL and GH.
